# Salivary hormone concentrations and technical-tactical performance indicators in beach volleyball: Preliminary evidence

**DOI:** 10.3389/fspor.2022.830185

**Published:** 2022-07-28

**Authors:** Yago Costa, Jarbas Domingos-Gomes, Franziska Lautenbach, Lawrence Hayes, Fabio Nakamura, Jefferson Lima, Lúcio Castellano, Gilmário Batista

**Affiliations:** ^1^Department Physical Education, Federal University of Paraíba, João Pessoa, Brazil; ^2^Department of Sport Psychology, Institute of Sport Science, Humboldt-Universität zu Berlin, Berlin, Germany; ^3^Institute for Clinical Exercise and Health Science, University of the West of Scotland, Paisley, United Kingdom; ^4^Technical School of Health, Federal University of Paraíba, João Pessoa, Brazil

**Keywords:** exercise physiology, exercise psychology, young athletes, match analysis, winner effect

## Abstract

The present study aimed to investigate (i) differences in salivary testosterone and cortisol concentrations before, during, and after simulated beach volleyball match, depending on match outcome (winning vs. losing); (ii) the relationship between technical-tactical performance indicators in beach volleyball and salivary hormonal concentrations (i.e., testosterone, cortisol). We hypothesized (i) salivary testosterone concentrations would be greater in winners and salivary cortisol would be lower; (ii) testosterone would associate with positive technical-tactical performance and cortisol would associate with negative technical-tactical performance. Sixteen athletes participated in the study and were grouped according to the result of a simulated game (winners: *n* = 8; losers: *n* = 8). Salivary hormone concentration of testosterone and cortisol were measured by enzyme-linked immunosorbent assay (pre-match, post first set, and post-match), and the coefficient of performance and efficiency were used as technical-tactical performance indicators. Regarding testosterone, there was a large effect size for match outcome after the first set (i.e., Winner vs. Losers) and a moderate effect size for the time in winners (pre-match vs. post-match). Regarding cortisol, there was a moderate effect size of time in losers only (pre-match vs. post-match). Moreover, cortisol pre-match was negatively correlated with the offensive performance (attack performance coefficient: *r* = −0.541; *p* = 0.030; attack efficiency: *r* = −0.568; *p* = 0.022). In conclusion, the effect of match outcome on testosterone and cortisol levels was moderate in winners and losers, respectively. Moreover, resting cortisol concentration appears to be related to a diminished attack technical-tactical performance. However, larger confirmatory studies are required to confirm these data to corroborate winning increases testosterone levels and/or reduces cortisol in a sporting setting.

## Introduction

Beach volleyball is an intermittent sport that alternates between effort and rest periods (Medeiros et al., 2014a; Costa et al., 2021). Players use jumps in various actions (e.g., spike, block), move small distances (Cortell-Tormo et al., 2011; Medeiros et al., 2014a), and can be classified as an “open skill sport,” because most actions are dynamic and unpredictable (Wang et al., 2013), which requires cognitive effort for satisfactory performance (Verburgh et al., 2014; Yu et al., 2019). Moreover, game actions can be classified as “continuous actions” (i.e., serve reception, set, and dig), where effectiveness is determined by keeping the ball in play; or “terminal actions” (i.e., serve, attack, and block), in which effectiveness is determined by winning the rally (Palao et al., 2015). It has been observed that serve and attack effectiveness determines the winner of the set (Michalopoulou et al., 2005) and is the best predictor of victory (Grgantov et al., 2005). Subsequently, in a more robust study with world-class U-19 and U-21 athletes, the dig, attack, and counter-attack performance coefficient exerted a substantial influence on determining victory (Medeiros et al., 2017). Yet, these studies are limited as they observed the match and determined performance indicators which determine set victory *a priori*. Bangsbo (2015) proposed a holistic model of determinants of sports performance, citing technical, tactical, and physiological characteristics which dictate sporting success. Resultantly, testosterone and cortisol are two important biomarkers frequently investigated within a sporting context, because they exhibit relationships with physical and psychological factors that are associated with technical-tactical performance (Thorpe and Sunderland, 2012; Cook and Beaven, 2013; Hayes et al., 2015a).

Acute salivary testosterone and cortisol response to exercise bouts have received substantial attention in recent years (Hayes et al., 2016). Meta-analytical evidence suggesting salivary testosterone is increased following aerobic and resistance type exercise (Hayes et al., 2015a). Similarly, salivary cortisol is increased following aerobic and resistance type exercise, but aerobic exercise produces a larger increase in salivary cortisol compared to resistance exercise. Likewise, salivary cortisol is also increased more than salivary testosterone in response to aerobic exercise (Hayes et al., 2015b). With regards to both *serum* hormones, the time course of peak increase following exercise is generally considered to be in the region of 5-20 min (West et al., 2010; Sellami et al., 2018), which is consistent with consistent with the peak in *salivary* steroid hormones examined herein (Hayes et al., 2014, 2015a).

Concerning testosterone, this steroid hormone is secreted by the interstitial cells of the testicles (leydig cells) and is regulated by the hypothalamic-pituitary-gonadal axis [HPG axis (Oyola and Handa, 2017)]. Some authors have suggested a connection between salivary testosterone and muscle power production and resistance in intermittent tasks (Moreira et al., 2013b), a fundamental physical characteristic of beach volleyball. These authors suggested that salivary testosterone explained 32% of the variance in vertical jump and 21% variance in the Yo-Yo intermittent endurance test. Moreover, testosterone has been associated with the neuroendocrinology model in competitive contexts. Mazur (1985) proposed the Biosocial Model of Status, which associated changes in testosterone concentration with the outcome of the competition (for a detailed review of the social neuroendocrinology of testosterone see Carré and Olmstead, 2015). This model proposes victory increases testosterone levels in winners, which has already been observed after challenges with high (Jiménez et al., 2012; Carré et al., 2013) and low (Mazur et al., 1992) physical demand. However, it is worth noting this model was guided by observations in male rhesus monkeys (Rose et al., 1972, 1975) and humans may display disparate patterns due to more complex societal structures. Moreover, testosterone is associated with dominance and social status (e.g., superiority over someone), but determining causality in this relationship is problematic (i.e., whether testosterone causes these behaviors or if the situation is responsible for the increase in testosterone) (Mazur and Booth, 1998).

Cortisol is under hypothalamic-pituitary-adrenal (HPA) axis control, and is secreted in response to acutely stressful situations associated with fight-or-flight behavior (Russell and Lightman, 2019). Therefore, during exercise, it is common for cortisol to increase (Hayes et al., 2015a), but the competitive level and the importance of competition seem to moderate the response (Slimani et al., 2017). It is reasonably well known that maladjusted cortisol levels seem to compromise cognitive function [e.g., working memory, cognitive flexibility, and interference control (Shields et al., 2016)]. Specifically in relation to sporting scenarios, cortisol decreased decision-making performance of jockeys (Landolt et al., 2017), impaired memory, and visual attention of junior orienteering athletes (Robazza et al., 2018), and compromised the technical-tactical performance of tennis players (Lautenbach et al., 2015) and golf score (Doan et al., 2007). In an investigation with badminton players, an increase in cortisol and a decrease in testosterone after losing was observed (Jiménez et al., 2012), in line with the Biosocial Model of Status. Moreover, negative performance outcomes may be associated with the anxiety experienced concomitantly with increased cortisol, and a link between cortisol, anxiety, and worsening performance in sports that require high precision has been repeatedly observed (Lim, 2018; Park et al., 2020).

Edwards and Kurlander (2010) demonstrated an increase in testosterone and cortisol after volleyball matches. Moreover, technical-tactical performance indicators have been well established for beach volleyball (Medeiros et al., 2017; Palao et al., 2018). However, the salivary cortisol and testosterone effect of victory in these athletes and the relationship with performance indicators remains unclear. Recently, Sansone et al. (2019), investigated if small-sided basketball game were useful in preparing athletes for endocrine responses. It is important to investigate the endocrine responses of training means (e.g., simulated games), because these mimic official competitions, and allow players to develop psychological skills such as coping apparatus (Slimani et al., 2017). Therefore, the present study aimed to (i) examine differences in testosterone and cortisol concentration before, during, and after a simulated beach volleyball match, depending on match outcome (i.e., winners vs. losers); (ii) examine the relationship between technical-tactical performance indicators (e.g., coefficient of performance and efficiency) in beach volleyball and salivary hormone concentrations. We hypothesized *a priori* that testosterone would be higher in winners before, during, and after the match in comparison to losers. Further, we expected cortisol to be higher in losers before, during, and after the match in comparison to winners. Finally, we hypothesized positive relationships between technical-tactical performance parameters such as attack and attack post-dig coefficient of performance and efficiency with salivary testosterone would exist, and negative associations would exist between salivary cortisol and technical-tactical performance.

## Materials and methods

### Participants

Sixteen young male beach volleyball athletes (winners: M_age_ = 16.9 ± 2.7 years; M_height_ = 1.86 ± 0.03 m; M_mass_ 79.8 ± 10.7 kg; M_BMI_ 22.9 ± 0.9 kg/m^2^; losers: M_aged_ 17.4 ± 2.8 years; M_height_ 1.82 ± 0.07 m; M_bodymass_ 72.7 ± 7.3 kg; M_BMI_ 21.8 ± 1.1 kg/m^2^; no difference between groups) who competed on the National Circuit organized by the Brazilian Volleyball Federation for at least 2 years, participated in the study. Participants trained beach volleyball skills 4.4 ± 1.2 days·week^−1^ in two training centers and completed strength and conditioning ~3 days·week^−1^. All athletes participated voluntarily, with the research team having invited participants based on the state ranking (eight best teams) to take part. The procedures were in line with the Declaration of Helsinki and approved by the Ethics Committee on Research with Humans, reference number 2.251.594. Athletes provided informed consent, and legal guardians of athletes under 18 years of age provided ascent.

### Design

In a simulated competitive setting, eight teams (16 players) participated in a match against a randomized opponent following the rules of the International Volleyball Federation (FIVB). Matches were officiated by the same referee with experience in national competitions. Official courts used were on the beach, and three Mikasa^®^ VLS300 balls with standard pressure of 200 mbar were used. All matches started at 3 p.m. to control circadian rhythmicity (Westermann et al., 2004; e.g., Hayes et al., 2010)on different days with similar weather conditions (temperature ~30°C) that players are accustomed to playing in.

Participants were instructed to not perform training sessions or consume alcohol 24h before data collection. Consumption of energy drinks or caffeinated beverages was prohibited on the day of data collection. Moreover, 30 min prior to salivary samples, eating and teeth brushing were prohibited. This information was attested through a checklist completed before the beginning of the collection procedures (Hayes et al., 2016). Saliva was collected before a 10 min warm-up, after the first set, and 10 min after the match. To ensure maximum effort, the winning team received an award for victory (i.e., sports equipment). Researchers involved in saliva collection and coaches (without giving instructions) watched the games to promote a competitive atmosphere (Parmigiani et al., 2009; Lautenbach et al., 2016).

### Measurements

#### Match outcome

Athletes were divided into winners and losers based on the outcome of the match. Thus, the team that won two sets (2-0 or 2-1) was declared the winner. Moreover, to win the set it was necessary to win at least 21 points with more than 2 points of difference from the opponent (e.g., 21 × 18), if necessary more points were played until one of the teams had a difference of two points (e.g., 24 × 22) following the Official Beach Volleyball Rules (FIVB). In total, there were four winning teams (eight athletes) and four losing teams (eight athletes). Matches were ~30 min in duration (match 1 = 29.4 min; match 2 = 31.1 min; match 3: 29.1 min; match 4: 33.2 min) with an average of 71 ± 6.8 rallies per match.

#### Technical-tactical performance

Matches were filmed using a camera (Sony^®^ DSC-SX21, Manaus, Brazil), supported on a tripod approximately 10 m from the end line, with a view of the entire playing space. Technical-tactical performance analysis was performed by two raters with experience in beach volleyball. The primary observer (A) was an Olympic Champion Coach with more than ten years of experience in the sport; the secondary observer (B) was a coach with more than five years of experience. Thus, independent observers “A” and “B” completed match analysis of all matches, categorizing actions using Lince^®^ 1.3 software. After 15 days, technical-tactical performance ratings were performed again to ensure robust intra-rater reliability. The intra-rater (i.e., day 0 and day 15) and inter-rater reliability (i.e. between rater “A” and “B”) can be considered “strong” to “almost perfect” (Cohen's kappa ≥ 0.80; McHugh, 2012), suggesting our data were reliable.

Actions were categorized into terminal actions and continuous actions. In detail, terminal actions were coded with scores “0” for error actions (e.g., hit in the net); “1” for maximum attack options for the opponent (e.g., allowed to build an organized attack with all the options—easy dig); “2” for limited attack options for the opponent (e.g., allowed to build an organized attack with limited options—moderate dig); “3” for no attack options for the opponent (e.g., did not allow to build an organized attack—dig without attack options); “4” for point actions for server, attack, block, and attack post-dig. For continuous actions “0”—error actions; “1”—no attack options; “2”—limited attack options; “3”—maximum attack options for serve reception, set and dig (continuous actions) (Palao et al., 2015). Finally, the coefficient of performance and efficiency was calculated using the equations 1 and 2 (Coleman, 2002). These performance indicators allow for more complete information because consider all actions adjusted for a total of attempts (Marcelino et al., 2010), and showed a moderate to large effect in determining the set result in U-19 and U-21 beach volleyball competition (Medeiros et al., 2017). In this way, the higher the score for terminal and continuous action, the better the technical-tactical performance.

##### Equation 1: Equation to calculate coefficient of performance


$$\begin{array}{lll} CP^{1} & = & \frac{\left(“x^{\prime \prime }\;scores\;1\right)+\left(“x^{\prime \prime }\;scores\;2*2\right)+\left(“x^{\prime \prime }\;scores\;3*3\right)+\left(“x^{\prime \prime }\;scores\;4*4\right)\;}{\sum actions\;all\;scores}\\ CP^{2} & = & \frac{\left(“x^{\prime \prime }\;scores\;1\right)+\left(“x^{\prime \prime }\;scores\;2*2\right)+\left(“x^{\prime \prime }\;scores\;3*3\right)\;}{\sum actions\;all\;scores} \end{array}$$

CP^1^ = coefficient of performance—terminal actions; CP^2^ = coefficient of performance—continuous actions; X = number of actions.

##### Equation 2: Equation to calculate efficiency


$$\begin{array}{l} EFF=\frac{\left(“x^{\prime \prime }\;scores\;4\;-\;“x^{\prime \prime }\;scores\;0\right)*100}{\sum actions\;all\;scores} \end{array}$$

EFF = efficiency; X = number of actions.

#### Salivary hormone analysis

Athletes were asked to provide 1 ml of saliva into plastic tubes without receiving any salivation stimulus (e.g., gum) to facilitate salivation. Participants used a straw, similar to the procedures of Lautenbach et al. (2015), and provided samples via passive drool as suggested by the Hayes et al. (2016) guidelines for practitioners and researchers. Saliva was immediately stored at −20°C until analysis. Salivary testosterone and cortisol levels were assayed in duplicate by enzyme-linked immunosorbent assay (ELISA) using commercial kits (DRG^®^ International; Frauenbergstr, Germany. Ref. SLV-3013 and Diametra^®^; Via Pozzuolo, Italy. Ref. DKO020 respectively). The absorbance readings were measured on the GloMax^®^-Multi microplate reader (Promega, California, United States, Ref. E7061). The inter and intra-assay coefficient of variation was <5% in all instances. Salivary samples were analyzed at the Laboratory of the Technical School of Health of the Health Sciences Center of the Federal University of Paraíba, following manufacturer's guidelines. Researchers that performed the biochemical analyses were blinded to the outcome of the matches.

### Statistical analysis

Data are presented as means and standard deviation, and all variables were normally distributed when tested by Shapiro-Wilk. Data were divided according to match outcome (winners vs. losers), so technical-tactical performance was compared using an independent *t*-test. Salivary hormones (testosterone and cortisol) were analyzed by two-way repeated-measures analysis of variance (ANOVA; time x match outcome). Subsequently, one-way ANOVAs with *a posteriori* t-tests and Bonferroni corrections were conducted to examine differences between the match stage (pre-match, after the first set, and post-match) and *a posteriori* t-test with Bonferroni correction was conducted to determine differences between winners and losers at a given match stage. Effect size (ES) for ANOVA is presented as partial eta squared ($\eta_{\text{p}}^{2}$) and interpreted as 0.01 as small, 0.09 as medium, and 0.25 as large (Suppiah et al., 2016; Mesquita et al., 2019). ES for pairwise caparisons is presented as Cohen's (d) (Cohen, 1988), adopting the magnitude <0.2 (trivial), 0.2 to 0.6 (small), >0.6 to 1.2 (moderate), >1.2 to 2 (large), 2 to 4.0 (very large) and >4 (almost perfect) (Hopkins et al., 2009). Furthermore, Pearson correlation coefficient (r) between technical-tactical performance and hormonal levels was calculated, with magnitude classified as 0 to 0.29 (small), >0.29 to 0.49 (moderate), >0.49 to 1 (large) (Cohen, 1988). All procedures were performed using IBM SPSS Statistics for Windows, Version 20.0 (Armonk, NY: IBM Corp.). Alpha levels are reported as exact P values, and we do not define *P* values as 'significant' or 'non-significant' as advised by the American Statistical Association (Hurlbert et al., 2019). Finally, we calculated the smallest worthwhile change of each hormone using the equation of Hopkins equation [smallest worthwhile change = 0.2 · standard deviation, whereby the 0.2 value is derived from the small effect size (Hopkins, 2004)]. Data are presented a mean ± standard deviation (SD).

## Results

### Technical-tactical key components of volleyball performance

The technical-tactical performance indicators are shown in Table 1. Winners and losers differed in attack post-dig performance coefficient (*p* = 0.001, *d* = 1.483, large effect) and efficiency (*p* = 0.021, *d* = 1.304, large effect), suggesting that, in the simulated matches in this study, these were the key technical-tactical performance indicators. Some indicators showed moderate differences such as attack performance coefficient (*p* = 0.084, *d* = 0.921) and efficiency (*p* = 0.098; *d* = 0.827), and were thus, interpreted as secondary to performance.

**Table 1:** Mean and standard deviation of technical-tactical performance indicators as a function of the set outcome.

	**Winner**		**Loser**			**ES**
**Action**	**M**	**SD**	**M**	**SD**	**Sig**.	**d**
**PC sever**	1.55	0.26	1.34	0.25	0.118	0.823 (moderate)
**PC server reception**	2.28	0.21	2.20	0.32	0.536	0.295 (small)
**PC set**	2.43	0.11	2.39	0.31	0.748	0.171 (trivial)
**PC attack**	2.49	0.64	1.98	0.45	0.084	0.921 (moderate)
**PC block**	2.10	1.35	2.23	1.68	0.871	0.085 (trivial)
**PC dig**	1.95	0.39	1.70	0.47	0.270	0.578 (small)
**PC set PD**	2.30	0.33	2.29	0.32	0.927	0.030 (trivial)
**PC attack PD**	2.54	0.82	1.51	0.54	0.010*	1.483 (large)
**EFF. Attack**	29.17	32.59	6.01	17.28	0.098	0.827 (moderate)
**EFF. Attack PD**	34.08	36.42	−9.64	30.32	0.021*	1.304 (large)

M, mean; SD, standard deviation; PC, performance coefficient; PD, post dig; EFF, Efficiency; ES, effect size. *p ≤ 0.05.

### Match outcome and hormonal concentration

**Figure 1:**
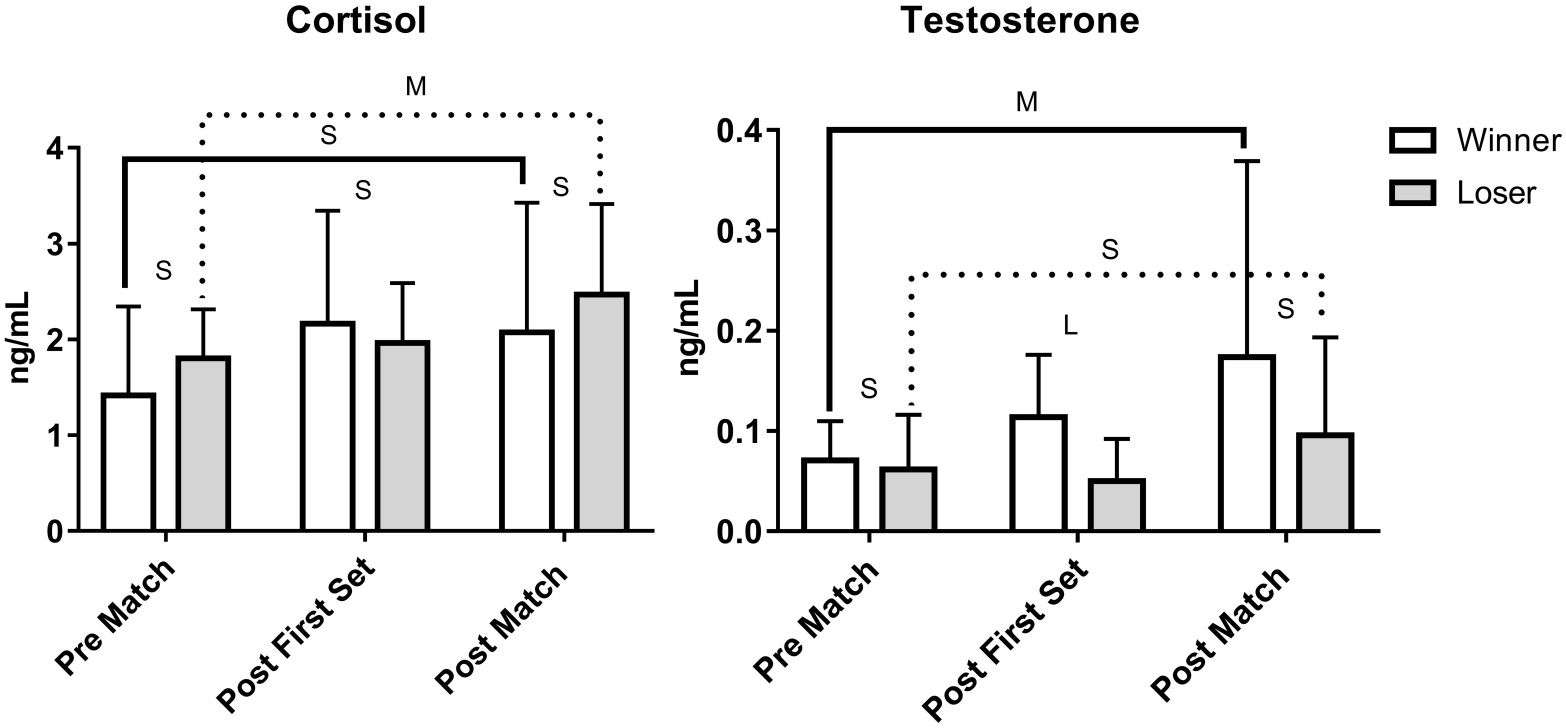
Effect size of match on cortisol and testosterone in winners and losers of a beach volleyball match. S, small effect size; M, moderate effect size; L, large effect size.

The ANOVAs displayed large main effects for time for both hormones, medium and small effects for match outcome for testosterone and cortisol respectively, and small and medium effects for the interaction for testosterone and cortisol respectively (see Table 2 for details). The increase in salivary testosterone in the winners from pre-match to post-first set and post-match exceeded the smallest worthwhile change, whilst the changes in losers did but only from pre- to post-match. The effect sizes by time and outcome are shown in Figure 1, and a large effect size was noted for testosterone between winners and losers after the first set whilst a moderate time effect was observed within winners from pre- to post-match. For cortisol, moderate effects were evident in losers from pre- to post-match. The increase in salivary cortisol in both winners and losers from pre-match to post- first set and post-match exceeded the smallest worthwhile change.

**Table 2:** Comparison of hormonal concentration between winners and losers.

	**Testosterone (ng/ml)**					**ANOVA**
	Pre-match	Post first set	Post-match	SWC	**Time**	*F*_(1.117.77)_ = 4.380; *p* = 0.068; $\eta_{\text{p}}^{2}$ = 0.385; power: 0.468
Winner	0.07 ± 0.04	0.11 ± 0.06	0.17 ± 0.20	0.008	**Outcome**	F_(1.007.00)_ = 1.727; *p* = 0.230; $\eta_{\text{p}}^{2}=$ 0.198; power: 0.207
Loser	0.06 ± 0.05	0.04 ± 0.04	0.09 ± 0.10	0.010	Time*Outcome	*F*_(2.0014.00)_ = 0.539; *p* = 0.595; $\eta_{\text{p}}^{2}$ = 0.071; power: 0.122
						**ANOVA**
	**Cortisol**_ng/ml_					
	Pre match	First set	Post-match	SWC	**Time**	*F*_(1.218.53)_ = 4.860; *p* = 0.052; $\eta_{\text{p}}^{2}=$ 0.410; power: 0.536
Winner	1.41 ± 0.93	2.16 ± 1.18	2.07 ± 1.36	0.186	**Outcome**	*F*_(1.007.00)_= 0.153; *p* = 0.707; $\eta_{\text{p}}^{2}$ = 0.021; power: 0.063
Loser	1.80 ± 0.51	1.96 ± 0.62	2.46 ± 0.95	0.102	Time*Outcome	*F*_(1.208.42)_ = 2.000; *p* = 0.195; $\eta_{\text{p}}^{2}$ = 0.222; power: 0.256

Mean ± standard deviation. SWC, Smallest worthwhile change.

### Relationships between technical-tactical key components of volleyball performance and hormonal concentration

Table 3 shows the correlations between hormone concentrations and technical-tactical performance (key indicators). Salivary cortisol pre-match was negatively correlated with attack performance coefficient (*r* = −0.541; *p* = 0.030), and efficiency (*r* = −0.568; *p* = 0.022) when participants were pooled. No other meaningful correlations were observed (see Table 3 for details).

**Table 3:** Correlation between technical-tactical key components of volleyball performance with hormonal analyses.

**Technical-tactical indicators**	**Pre-match**	**Post first set**	**Post-match**	
**PC attack**	−0.049 (0.856)	0.343 (0.194)	0.144 (0.595)	**Testosterone**
**PC attack PD**	−0.231 (0.390)	0.209 (0.437)	0.335 (0.205)	
**EFF. attack**	−0.099 (0.717)	0.266 (0.320)	−0.076 (0.779)	
**EFF. attack PD**	−0.097 (0.721)	0.120 (0.657)	0.253 (0.344)	
**PC attack**	−0.541 (0.030)^*^^L^	−0.210 (0.436)	−0.267 (0.328)	**Cortisol**
**PC attack PD**	−0.218 (0.416)	−0.147 (0.587)	−0054 (0.841)	
**EFF. attack**	−0.568 (0.022)^*^^L^	−0.185 (0.493)	−0.289 (0.277)	
**EFF. attack PD**	−0.209 (0.438)	−0.213 (0.428)	−0.091 (0.737)	

PC, performance coefficient; PD, post-dig; EFF, efficiency; r (p).

^L^ large correlation.

* p < 0.05.

## Discussion

This study aimed to analyze (i) differences in salivary testosterone and cortisol concentrations before, during, and after a simulated beach volleyball match, depending on outcome; (ii) the relationship between technical-tactical performance indicators in beach volleyball and hormonal concentrations. Results from the present investigation indicate that salivary testosterone was higher in winners after the first set, and after the match in comparison to losers. However, there were no differenced pre-match. The difference between winners' and losers' salivary cortisol was small pre-match, after the first set, and post-match. The losers' increase in salivary cortisol from pre- to post-match was identical to that of winners (mean increase = 0.66 ng/ml) but resulted in a greater effect size due to a smaller standard deviation. Thus, we suggest this difference in magnitude is likely artefactual. Our final hypothesis was that positive relationships between technical-tactical performance parameters and salivary hormones would exist. There were two meaningful correlations between salivary cortisol and attack performance and salivary cortisol and efficiency and so we can partly accept out hypothesis, however no other correlations were observed which suggests there is some ambiguity about the predictive potential of salivary hormones for technical-tactical performance in beach volleyball.

To the best of our knowledge, this is the first study to assess these variables in beach volleyball players. Salivary hormone concentrations were hypothesized to increase due to physical and psychological arousal throughout the match (Kraemer and Ratamess, 2005; Aceña, 2020; Mendoza et al., 2020). However, this effect should depend upon social status and physical demand. For example, young volleyball starters (i.e., players who starting the match playing), increased pre- to post-match testosterone and cortisol levels, but this effect was not observed in reserve players (Edwards and Kurlander, 2010). This could be resultant from two factors, 1; reserves did not exert as much physical effort as starters due to reduced playing time, or 2: the reduced social status of being considered a reserve player. In male handball athletes, a large effect of the match was observed on testosterone and moderate effect for cortisol, but unfortunately the authors did not present the results in relation to the outcome of the match (Foretic et al., 2020). As such, separating the effect of match outcome from physical effort is difficult from these previous studies. In the present investigation however, we observed a similar effect on salivary cortisol between winners and losers, but winners increased salivary testosterone to a larger effect than losers. This is in line with a meta-analysis which reported salivary cortisol increased greatly following exercise, and more so to aerobic exercise than resistance exercise (i.e., a greater physical effort and metabolic demand; Hayes et al., 2015a). Therefore, we speculate that salivary cortisol is most sensitive to physical effort, and as both winners and losers in the present study completed the same physical effort, their salivary cortisol responses were near-identical. Conversely, salivary testosterone increases to a lesser degree than salivary cortisol following exercise of all types (Hayes et al., 2015a), but is known to increase following victory, even in the absence of physical effort (van der Meij et al., 2012). Therefore, we speculate that salivary testosterone is most sensitive to match outcome, and as both winners and losers in the present study completed the same physical effort, but disparate match outcomes resulted in different salivary testosterone concentrations, in line with the biosocial theory (Mazur, 1985).

This speculation is supported by Oliveira and colleagues who reported a positive change (i.e., delta pre- to post-match) in testosterone in female soccer players but only in winners (Oliveira et al., 2009). These authors however, proposed an alternative explanation to us, and suggested results observed may be due to more physical effort by winners. Whilst it is known that salivary testosterone increases following high-intensity exercise (Leal et al., 2021) and modification of tactical tasks can result in different intensities, and consequently change testosterone responses (Sansone et al., 2019), we would expect that in the present study, differences in exercise intensities and/or volumes would be reflected by changes in salivary cortisol. Supporting our supposition, Fry et al. (2011) reported the greatest increase in testosterone in wrestlers following winning. In the present study, the greatest effect size between winners and losers (i.e., large effect) for salivary testosterone was observed after the first set. The biosocial theory (Mazur, 1985) can explain this effect, as winners' salivary testosterone was increased following a successful confrontation. This theory proposes winning promotes a feeling of dominance and encouragement for future confrontations. Conversely, loss provokes feelings of submission and avoidance of further confrontation. This is particularly important for beach volleyball, because matches in competition are played on the same or successive days, which reduces the time to regain courage. Moreover, it is common throughout a season to face the same opponent repeatedly during tour events.

In facing the physical stress of a match, homeostasis is disturbed. In response, the autonomic nervous system is activate, mainly through sympathetic branches (Rotenberg and McGrath, 2016). Then, because of the HPA axis, cortisol is secreted, the internal organs (e.g., heart) and cognitive functions (e.g., attention) are stimulated (Rotenberg and McGrath, 2016; Anderson et al., 2019). In an appropriate stress response, cortisol is sufficient to meet the challenge, increase energy, and improve decision making and motor control through enhanced arousal. Regardless of match outcome, salivary cortisol was increased during the match in the present study. Moreover, losers had ~28% more salivary cortisol that winners (win: 1.41 ± 0.93 vs. 1.80 ± 0.51 ng/ml) pre-match, which exhibited negative correlations with performance indicators (coefficient of performance and attack efficiency). Previously, cortisol was higher in losing tennis players before, during, and after the match and negatively correlated with performance parameters (Lautenbach et al., 2015). Other studies have found a negative effect on golf (Doan et al., 2007) and orienteering (Robazza et al., 2018), for match score and executive functions, respectively. Perhaps the negative effect of excessive cortisol can be explained by arousing negative emotions that impair performance. Díaz et al. (2013) reported a relationship between cortisol and mood of swimmers, feelings of tension, anxiety and hostility experienced in response to the stress caused by competition were observed. Moreover, the anxiety provoked by cortisol can reduce precision in motor tasks (Lim, 2018; Park et al., 2020), and if the precision was attenuated too in beach volleyball players, this is potentially problematic, as the hit action, which is large responsible for the victory (Medeiros et al., 2017), is highly dependent on this skill. However, we must exert caution interpreting these small differences (1.41 ± 0.93 vs. 1.80 ± 0.51 ng/ml) as meaningful, given the large fluctuations and variation observed in salivary cortisol (Hayes et al., 2014, 2016), so whether these differences are biologically significant remains to be determined.

A positive correlation of performance indicators with salivary testosterone was hypothesized because spike attacks are more effective and are predictive of match outcome (Medeiros et al., 2014b), and testosterone is related to muscle mass. However, this was not observed, possibly because technical-tactical performance depends more on cognitive skill than attack power (Trecroci et al., 2021), and also our participants were anthropometrically similar. Conversely, male rugby players exhibited technical-tactical performance correlations with high testosterone and low cortisol responses (Cook and Crewther, 2012). Perhaps the nature of the modality (invasion sports vs. net sports) moderates the technical-tactical and hormonal relationship. Beach Volleyball is a sport without direct contact between players, whereas rugby is perhaps the team sports that most allows contact between players and aggressive behavior seems interesting. Based on the biosocial theory (Mazur, 1985), it is possible that losers are less motivated and submissive but since in volleyball the physical risk is lower, the physical conflict is less determining of match outcome. Moreover, our data was from a simulated match and in general official matches provoke more significant endocrine responses. Moreira et al. (2012) reported a cortisol variation of 108% pre- to post-basketball official match, and match importance moderate cortisol responses in elite young volleyball players (Moreira et al., 2013a). Future investigations could consider investigating official competitions and a greater number of athletes, like at an international tournament. Moreover, physical effort could be monitored by GPS and/or heart rate, as well as the level of stress through subjective scales (e.g., Daily Analysis of Life Demands in Athlete—DALDA; Moreira and Cavazzoni, 2009), since we suggest stress perception may be responsible for the impairment of technical-tactical performance. Moreover, investigations need to be carried out to assess whether exogenous changes in testosterone and cortisol concentrations caused by the use of WADA-permitted substances (e.g., caffeine) or exercise (e.g., strength training session) effect on psychological and technical-tactical performance.

### Practical application

Success in beach volleyball does not depend only on technical-tactical performance, but mental skills fundamental to the result, corroborating the holistic model of the determinants of sports performance (Bangsbo, 2015). Simulated matches appear useful in investigating athletes for endocrine responses, but it is imperative coaches must create a very real scenario (e.g., referee, reward). Despite the preliminary nature of data presented here (as matches were simulated), we found a relationship between pre-competition salivary cortisol and technical-tactical performance. Therefore, it is important that coping strategies (i.e., breathing techniques, self-talk, and etc.) are adopted to manage stress (Kurimay et al., 2017), especially with young athletes. This may be even more pertinent during tournament play where pre-competition arousal and stress may be even greater. Coaches and sport psychologists have previously adopted motivational strategies (e.g., positive feedback and reviewing tape recordings of one's previous best performances). These strategies increased salivary testosterone, lowered salivary cortisol, and resulted in better performance (Cook and Crewther, 2012), which could contribute to match outcomes in beach volleyball.

Finally, the endocrine responses presented are the result of endogenous changes as a consequence of the stress of competition and exercise (i.e., simulated match). The use of substances which promote alterations in these markers is strongly discouraged when prohibited by the World Anti-Doping Agency (WADA). Furthermore, a WADA-permitted strategy to increase salivary testosterone is strength training (Beaven et al., 2008), but finding the balance between post-activation potentiation of salivary testosterone and fatigue could be problematic, and any positive effects on match outcome may be artefactual, and not endocrine-related at all.

### Limitation of the study

This study has two main limitations, which are the simulated matches and the small sample size. Competition seriousness and competition level modulate testosterone and cortisol responses in soccer players (Jiménez et al., 2020), and the same is expected with beach volleyball athletes. However, in the present study, only winners received an award, which may have created an atmosphere like actual competition. In this context, we still observed a difference in salivary testosterone between winners and loser, so believe our method was robust in producing the emotional state of success and loss. Concerning sample size, our relatively small sample was necessary to maintain the technical-tactical quality as all athletes were at national level. We feel this justifies our statistical approach (i.e., focusing on effect size rather than alpha values), as statistical power observed for the ANOVAs was <0.80, allowing a type II error when rejecting an existing effect (Field, 2009). This was unavoidable as our *a priori* intention was to focus on elite athletes only, and thus an *a priori* power calculation would have been purely academic, as we only had the ability to recruit 16 participants. Finally, we used the immunoassay method to measure hormone levels, although this method presents a good correlation with the gold standard of evaluation (i.e., mass spectrometry), this method is known to have limitations, particularly with quantification of salivary steroid hormones (Granger et al., 2004; Hayes et al., 2014, 2015b,c; Fiers and Kaufman, 2015).

## Conclusion

In conclusion, our data tentatively suggest salivary testosterone is capable of differentiating winners from losers with winners experiencing greater salivary testosterone concentrations, but salivary cortisol is likely not. Salivary cortisol appears more sensitive to physical exertion than match outcome, but there was a small difference pre-match between winners and losers. Pre-match salivary cortisol appears to be inversely related to technical-tactical attack performance, so there may be an association between the pre-match anxiety or arousal and poorer technical-tactical attack performance. Finally, we suggest coaches can use simulated matches as a means of exposing athletes to competition scenarios, as evidenced by neuroendocrine responses congruent with winning and losing. This could develop coping skills of athletes to manage stress when it comes to competition match play.

## Data availability statement

Inquiries regarding the raw data supporting the conclusions of this article can be directed to the corresponding author.

## Ethics statement

The studies involving human participants were reviewed and approved by Ethics Committee with Human Beings of the Medical Sciences Center - Federal University of Paraíba (No. 2.251.594). Written informed consent to participate in this study was provided.

## Author contributions

YC, JD-G, and GB: study design, data collection, data analysis, data interpretation, and writing. FL and LH: data interpretation and writing. JL and LC: data collection, data analysis, and data interpretation. All authors contributed to the article and approved the submitted version.

## Conflict of interest

The authors declare that the research was conducted in the absence of any commercial or financial relationships that could be construed as a potential conflict of interest.

## Publisher's note

All claims expressed in this article are solely those of the authors and do not necessarily represent those of their affiliated organizations, or those of the publisher, the editors and the reviewers. Any product that may be evaluated in this article, or claim that may be made by its manufacturer, is not guaranteed or endorsed by the publisher.
